# Effects of supplemental feeding of Chinese herbal mixtures to perinatal sows on antioxidant capacity and gut microbiota of sows and their offspring piglets

**DOI:** 10.3389/fmicb.2024.1459188

**Published:** 2024-09-12

**Authors:** Xuelei Duan, Xiao Wang, Zhaonian Li, Chenggong Liu, Yongzhan Bao, Wanyu Shi, Xinghua Zhao

**Affiliations:** ^1^College of Traditional Chinese Veterinary Medicine, Hebei Agricultural University, Baoding, China; ^2^Hebei Provincial Veterinary Biotechnology Innovation Center, Baoding, China; ^3^Hebei Provincial Traditional Chinese Veterinary Medicine Technology Innovation Center, Baoding, China

**Keywords:** antioxidant capacity, gut microbiota, piglets, QZGSP, sows

## Abstract

The stress response of pig herds poses a significant challenge in the pig breeding industry, and investigating strategies to mitigate this stress is of paramount importance. The objective of this study was to investigate the impacts of supplemental feeding of Chinese herbal mixtures to perinatal sows on antioxidant capacity and gut microbiota of sows and their offspring piglets. A total of 60 healthy sows (Large white) at fourth parity were randomly assigned to five treatment groups. The control group received a basal diet, while the TRT1 group received a basal diet supplemented with 2kg/t Bazhen powder (BZP). The TRT2, TRT3, and TRT4 groups were fed a basal diet supplemented with 1kg/t, 2kg/t, and 3kg/t Qi-Zhu- Gui-Shao soothing liver and replenishing blood powder (QZGSP), respectively. The trial lasted for 5weeks, starting from day 100 of gestation until day 21 of delivery. The results demonstrated that the inclusion of 2kg/t and 3kg/t QZGSP significantly enhanced the antioxidant capacity of sows and their offspring piglets to different degrees, thereby effectively alleviating oxidative stress. Analysis of gut microbiota revealed that QZGSP influenced the composition of gut microbiota in both sows and their offspring piglets. Specifically, at the genus level, the abundance of *Christensenellaceae_R-7_group* in the gut microbiota of sows in the TRT4 group was significantly lower than that in the TRT1 group (*p* < 0.05), while the relative abundance of Lactobacillus in the gut microbiota of sows in the TRT4 group was significantly higher than that in the CON group (*p* < 0.05). Furthermore, at the genus level, compared to those in the TRT1 group, piglets from the TRT4 group exhibited a significant decrease in relative abundance of *Escherichia-Shigella*, Parabacteroides, and *Methanobrevivacter* (*p* < 0.05), but a significant increase in *Phascolarctobacterium* (*p* < 0.05). Spearman correlation analysis indicated a positive correlation between relative abundance of *Christensenellaceae_R-7_group* and serum contents of T-AOC and CAT (*p* < 0.05), as well as a negative correlation with serum concentration MDA (*p* < 0.05). Additionally, there was a positive correlation between relative abundance *Lactobacillus* and serum levels SOD (*p* < 0.01) and GSH-Px (*p* < 0.05). Therefore, supplementation of 3kg/t QZGSP in the periparturient sow diet significantly augmented antioxidant capacity in both sows and offspring piglets, while concurrently modulating the composition and structure of their intestinal microflora. The findings from this study demonstrate that QZGSP represents a beneficial feed additive for perinatal sows.

## 1 Introduction

The stress reaction of pig herds is currently a vital concern in the pig industry, as it poses a considerable threat to the health and well-being of pigs, which can result in significant financial losses. Modern intensive farming practices, early weaning techniques for piglets, and fence feeding for sows have been implemented to improve production efficiency in pig farms but have also induced significant stress reactions in pig herds. Meanwhile, sows commonly suffer reactive oxygen species (ROS) due to increased energy demands and metabolic burdens during late gestation and lactation (Tan et al., 2021; Yan et al., 2021), which do not fully recover until the weaning period (Berchieri-Ronchi et al., 2011), leading to a decline in reproductive performance, immune function, and lactation performance of sows. Consequently, this affects the growth, development, and health status of the fetus and offspring piglets (Flowers and Day, 1990; Zhao et al., 2013; Zhao and Kim, 2020). Weaning stress often leads to reduced feed intake, increased diarrhea rate, and growth retardation in weaned pigs (Tang et al., 2022). Therefore, improving the antioxidant capacity of pig herds is particularly important for optimizing pig production.

The utilization of Chinese herbal mixture (CHM) as a strategy to mitigate stress-induced damage in pig herds is a promising option, supported by the demonstrated beneficial effects of CHM on stress response. The utilization of Chinese herbs in livestock animals has a rich history spanning over 2000 years (Gong et al., 2014). As previously reported, dietary supplementation with a mixture herbal extract (*Scutellaria baicalensis and Lonicera japonica*) could improve the antioxidant capacity of sows at farrowing day (Wang et al., 2022). Dietary supplementation of a Chinese herbal medicine (CHM) containing *Paeonia lactiflora*, licorice, dandelion, and tea polyphenols has been shown to enhance serum antioxidant capacity and modulate the gut microbiota composition in weaning pigs (Xu et al., 2022). Wang M. et al. (2021) reported that the dietary supplementation with the herbal extract mixture can regulate the antioxidant capacity and modify the composition of colonic bacteria in weaning piglets. These findings confirm the potential of Chinese herbal mixture (CHM) in combating oxidative stress in pigs.

However, previous studies have little mentioned the use of perinatal sows dietary supplementation of Chinese herbal mixture (CHM) to mitigate the oxidant stress of sows and their offspring piglets, and modulate the composition and structure of gut microbiota in sows and their offspring piglets. In general, Chinese herbal medicine compounds contain multiple bioactive ingredients that synergistically enhance efficacy compared to using a single herb alone (Wang et al., 2008). However, the factors influencing the health status of sows and their offspring piglets are exceedingly intricate, so the efficacy of a singular Chinese herbal medicine in addressing these complex issues arising from sows and their offspring piglets is often limited (Keith et al., 2005). Bazhen powder (BZP) is a traditional nourishing formula, which contains *Codonopsis pilosula* (Franch.) Nannf., *Atractylodes macrocephala* Koidz., *Poria cocos* (Schw.) Wolf, *Angelica sinensis* (Oliv.) Diels, *Ligusticum chuanxiong* Hort, *Paeonia lactiflora* Pall., *Rehmannia glutinosa* (Gaertn.) DC., *and Glycyrrhiza uralensis* Fisch., currently utilized formula for treating diseases with deficiency of *qi* and blood (Lu et al., 2023). Qi-Zhu-Gui-Shao soothing liver and replenishing blood powder (QZGSP) is a self-prepared formula based on the syndrome of “*qi*-blood deficiency and Gan-*qi* stagnancy.” Its main ingredients are *Astragalus membranaceus* (Fisch.) Bunge, *A. macrocephala* Koidz., *A. sinensis* (Oliv.) Diels, *P. lactiflora* Pall., *Bupleurum chinense* DC. *P. cocos* (Schw.) Wolf. Studies have shown that *Astragalus,* as one of the traditional medicinal herbs, has polysaccharides, saponins and flavonoids as its main components, which have antioxidant properties (Yao et al., 2024). While as the active ingredients of *A. macrocephala* Koidz, Polysaccharides contain the antioxidant and gastrointestinal mucosa protection properties (Li et al., 2022). It has been reported that *A. sinensis* polysaccharide (ASP) is one of the main active components of *Angelica sinensis* (AS), with the with important pharmacological activities, such as anti-oxidation, hematopoietic, liver protection, and so on Jijuan et al. (2021). *Paeoniae Radix Alba*, a dried root of *P. lactiflora*, Pall., contains antioxidant, liver protection, and immunological modulation properties (Yang et al., 2024). Saikosaponins (SS), the principal bioactive constituent of *Bupleuri Radix*, exhibits robust antioxidant activity (Zeng et al., 2023). Polysaccharide derived from Poria cocos (PCP) represents a prominent bioactive constituent, exhibiting a wide range of biological activities including antioxidant, anti-inflammatory, and hepatoprotective effects (Xu et al., 2022). Chinese herbal mixture (CHM) has been widely adopted as the preferred choice for mitigating stress in pig industry due to their ability to modulate the oxidative stress, wide safety and availability. However, the exact mechanisms by which Chinese herbal mixtures exert their effects are not yet fully understood. In this study, Chinese herbal mixture (CHM) contain six herbs with antioxidative properties was selected to explore the mechanism of Chinese herbal mixture (CHM) in combating stress in pig stress, taking into account the changes in fecal microbiota. The results of study aimed to provide theoretical guidance for the application of Chinese herbal mixture (CHM) in pig production. Given the biological similarities between pigs and humans, our research has the potential to provide a scientific nutritional reference for perinatal mothers.

## 2 Materials and methods

The experimental procedures of this study received approval from the Animal Care and Use Committee of Hebei Agricultural University (approval No. 2022161). The animal experiments took place at Weijia great grandparent farm situated in Pingu district, Beijing, China.

### 2.1 Chinese herbal medicine mixture formula

The formula of Bazhen powder (BZP) used in this study consisted of *C. pilosula* (Franch.) Nannf, *A. macrocephala* Koidz, *P. cocos* (Schw.) Wolf, *A. sinensis* (Oliv.) Diels, *L. chuanxiong* Hort, *P. lactiflora* Pall, *R. glutinosa* (Gaertn.) DC, and *G. uralensis* Fisch, in a ratio of 1:1:1: 1:1:1: 1:1. The formula of Qi-Zhu-Gui-Shao soothing liver and replenishing blood powder (QZGSP) used in this study consisted of *A. membranaceus* (Fisch.) Bunge, *A. macrocephala* Koidz, *A. sinensis* (Oliv.) Diels, *P. lactiflora* Pall, *B. chinense* DC, and *P. cocos* (Schw.) Wolf, in a ratio of 6:3:3: 4:2:2. The above Chinese herbal medicine (CHM) mixture was commissioned to be processed and manufactured by Wuhan HVSEN Biotechnology Co., Ltd.

### 2.2 Animals, feeding and management

A total of 60 healthy pregnancy sows with fourth parity and the similar backfat thickness were selected. Then, they were randomly divided into 5 treatment groups. In the control group, the sows were fed a basal diet. In the TRT1 group, the sows were fed a basal diet supplemented with 2 kg/t BZP. In the TRT2, TRT3, and TRT4 groups were fed a basal diet supplemented with 1 kg/t, 2 kg/t, and 3 kg/t QZGSP, respectively. The trial lasted for 35 days, starting from day 100 of gestation until day 21 of delivery. The basic diet was formulated in accordance with the NRC (2012) guidelines to meet the nutritional requirements of sows. The composition and nutrient levels of the basal diet were presented in Supplementary Table S1.

During the period from day 100 to day 110 of gestation, pregnant sows were individually housed in stalls and provided with a controlled diet of 3 kg per day. On the 111th day of gestation, sows were transferred to individual farrowing pens equipped with an enclosed heated creep area. From this day until delivery, each sow received a daily allowance of 2.5 kg. sows did not receive any feed on the day of farrowing. On the first day after delivery, sows were fed a lactation meal twice a day (at 7:30 AM and 2:30 PM), starting with an intake of 2.5 kg/day and gradually increasing by 0.5 kg/day until *ad libitum* feeding was reached. Throughout the experiment, water was available *ad libitum* for all sows and suckling piglets. All sows were exposed to the same controlled growing environment where relative humidity and temperature were automatically regulated.

### 2.3 Sample collection

On days 0 and 21 of lactation, 6 sows per group were randomly selected for blood and fresh feces sampling. A 10 mL blood sample was collected via ear vein puncture using sterile vacuum tubes. Fresh feces were obtained by gently massaging the sow’s rectum and transferred into 2 mL freezer tubes, which were then stored in a liquid nitrogen tank until subsequent analysis.

On days 11 and 21 of lactation, blood samples were collected from a total of 12 piglets per group (selected from 6 sows, with 2 piglets chosen from each litter based on their proximity to the mean body weight for that particular litter). A sterile vacuum tube was used to collect a 10 mL blood sample via jugular vein puncture.

On day 21 of lactation, 6 piglets per group (the 6 piglets were from the same 6 piglets for blood collection) were euthanized humanely. The entire intestine was carefully removed from the abdominal cavity and ligated at each section junction. Aseptic collection of digesta samples from the proximal colon was performed using 2 mL freezing tubes, followed by immediate snap-freezing in liquid nitrogen and subsequent storage at – 80°C in a low-temperature refrigerator for further analysis.

All the blood samples in this study were left at room temperature for 30 min before being centrifuged at 3000 r/min at 4°C for 15 min to obtain serum. The separated serum was then refrigerated at −20°C for subsequent testing.

### 2.4 Determination of serum antioxidant capacity

The concentrations of total antioxidant capacity(T-AOC), superoxide dismutase (SOD), glutathione peroxidase (GSH-PX), catalase (CAT), and malonaldehyde (MDA) in serum, were measured by the commercial kits (Nanjing Jiancheng Bioengineering Institute, Nanjing, China) according to the manufacturer’s instructions.

### 2.5 DNA extraction, PCR amplification, 16S rRNA gene sequencing, and analysis

The Hipure Stool DNA Kit (Model D3141, Magen Biotechnology Co., Ltd., Guangzhou, China) was used by the manufacturer’s instructions to extract the microbial DNA from fecal samples of sows and colon chyme from piglets. A Nanodrop 2000 spectrophotometer (Thermo Fisher Scientific Inc., DE, United States) was used to determine the amount of DNA, and agarose gel electrophoresis was used to assess the integrity of the DNA. To amplify the V3-V4 region of the bacterial 16S rRNA gene, specific primers were employed (Forward: 5′-CCTAYGGGRBGCASCAG-3′, Reverse: 5′-GGACTACNNGGGTATCTAAT-3′). The amplicon probes (Beckman Coulter, Inc., United States) were used to purify the PCR products after they were seen on 2% agarose gels. The QuantiFluor TM-ST fluorometer (Promega, Madison, WI) was then utilized to quantify the results. The manufacturer’s instructions were followed to construct sequencing libraries using the Illumina DNA Prep Kit (Illumina, CA, United States). With Life Technologies’ ABI StepOnePlus Real-Time PCR System (Foster City, United States), the library’s quality was evaluated. We used the Novaseq 6,000 platform (Guangzhou GENE DENOVO Biotechnology Co., Ltd., Guangdong, China) for sequencing.

In this study, the FASTP (version 0.18.0) (Chen et al., 2018) software was used to control the quality of raw data obtained high quality clean reads. The FLASH (version 1.2.11) (Magoč and Salzberg, 2011) was used to overlap paired reads as raw tags, with a minimum overlap of 10 bp and 2% mismatch error rate. Referring to Qiime’s Tags quality control process, the raw tags were intercepted and filtered for long length to get high quality clean tags (Caporaso et al., 2010). The UPARSE (version 9.2.64) (Edgar, 2013) pipeline was used to cluster the clean tags into operational taxonomic units (OTUs) with a similarity of at least 97%. The UCHIME algorithm was used to eliminate all chimeric tags, yielding effective tags for additional study (Edgar et al., 2011). Within each cluster, a representative sequence was chosen based on the tag sequence with the highest abundance. The representative OTU sequences were taxonomically classified into organisms using a naive Bayesian model implemented in RDP classifier (version 2.2) (Wang et al., 2007) based on the SILVA database (version 138.1) (Pruesse et al., 2007), with a confidence threshold range of 0.8–1. The community composition of each sample was quantified at various species taxonomic levels.

### 2.6 Statistics and analysis

Excel software was used to compile and assemble the experiment data. GraphPad Prism 9.4.0 software (GraphPad software Inc., San Diego, CA, United States) was used to construct bar graphs, with data displayed as “mean ± standard deviation.” With *p* < 0.05 signifying statistical significance, the experimental results were presented as “mean ± standard deviation” after one-way analyses were carried out using IBM SPSS 26.0 software (SPSS Inc., Chicago, IL, United States). The correlation coefficients between floras and serum antioxidant indexes were analyzed using the Spearman statistical method. Furthermore, a *p*-value of less than 0.05 was considered statistically significant.

The 16S rRNA sequencing data of all samples were analyzed with the Omicsmart online platform.^1^ The QIIME (version 1.9.1) was used to calculated the alpha diversity, including Chao1, ACE, Shannon, Simpson (Edgar et al., 2011). Tukey HSD test was used to analyze the difference of Alpha diversity between groups.

Principal coordinate analysis (PCoA analysis) based on unweight_unifrac distance algorithm was used to test the similarity of microbial community structure among samples, and Adonis test based on unweight_unifrac distance algorithm was used to analyze whether the difference of microbial community structure among sample groups was significant.

## 3 Results

### 3.1 Effects of supplemental feeding of QZGSP to perinatal sows on serum antioxidant capacity in sows

**Figure 1:**
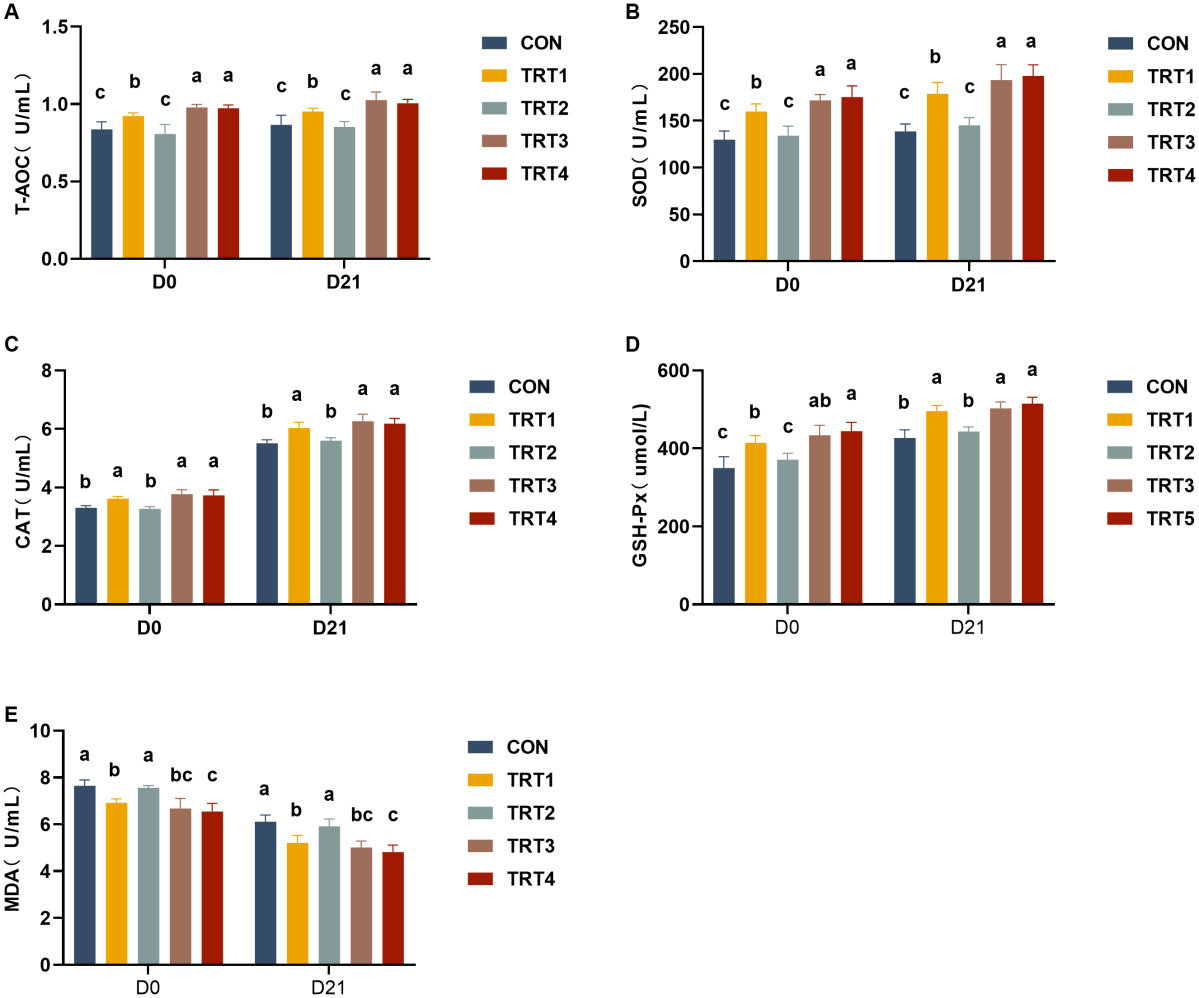
Effects of supplemental feeding of QZGSP to perinatal sows on serum antioxidant capacity in sows. Concentrations of serum T-AOC **(A)**, SOD **(B)**, CAT **(C)**, GSH-Px **(D)**, and MDA **(E)** among the five groups from sows on days 0 and 21 of lactation. Control (CON, basal diet), treatment group 1 (TRT1, basal diet +2 kg/t BZP), treatment group 2 (TRT2, basal diet +1 kg/t QZGSP), treatment group 3 (TRT3, basal diet +2 kg/t QZGSP), and treatment group 4 (TRT4, basal diet +3 kg/t QZGSP). The same lowercase letters indicate no statistically significant difference (*p* > 0.05), while different lowercase letters indicate a statistically significant difference (*p* < 0.05). The same to below.

The results of serum antioxidant capacity in sows are presented in Figure 1. As shown in Figures 1A,B, sows in TRT1, TRT3, and TRT4 groups exhibited significantly increase contents of serum T-AOC and SOD compared with the CON group on days 0 and 21 of lactation (*p* < 0.05), while the concentrations of serum T-AOC and SOD of sows in the TRT3 and TRT4 groups were significantly higher than those of the TRT1 group (*p* < 0.05). As depicted in Figures 1C,D, sows in TRT1, TRT3, and TRT4 groups demonstrated higher contents of CAT and GSH-Px than those observed in the CON group on days 0 and 21 of lactation (*p* < 0.05). In addition, when compared with the TRT1 group, sows in the TRT4 group showed a significant increase in GSH-Px contents on day 0 of lactation (*p* < 0.05). As shown in Figure 1E, compared with the CON group, sows in TRT1, TRT3, and TRT4 groups had significantly lower concentrations of MDA than that in the CON group on days 0 and 21 of lactation (*p* < 0.05); compared with the TRT1 group, the contents of MDA in TRT4 group were significantly reduced on days 0 and 21 of lactation (*p* < 0.05).

### 3.2 Effects of supplemental feeding of QZGSP to perinatal sows on serum antioxidant capacity in piglets

**Figure 2:**
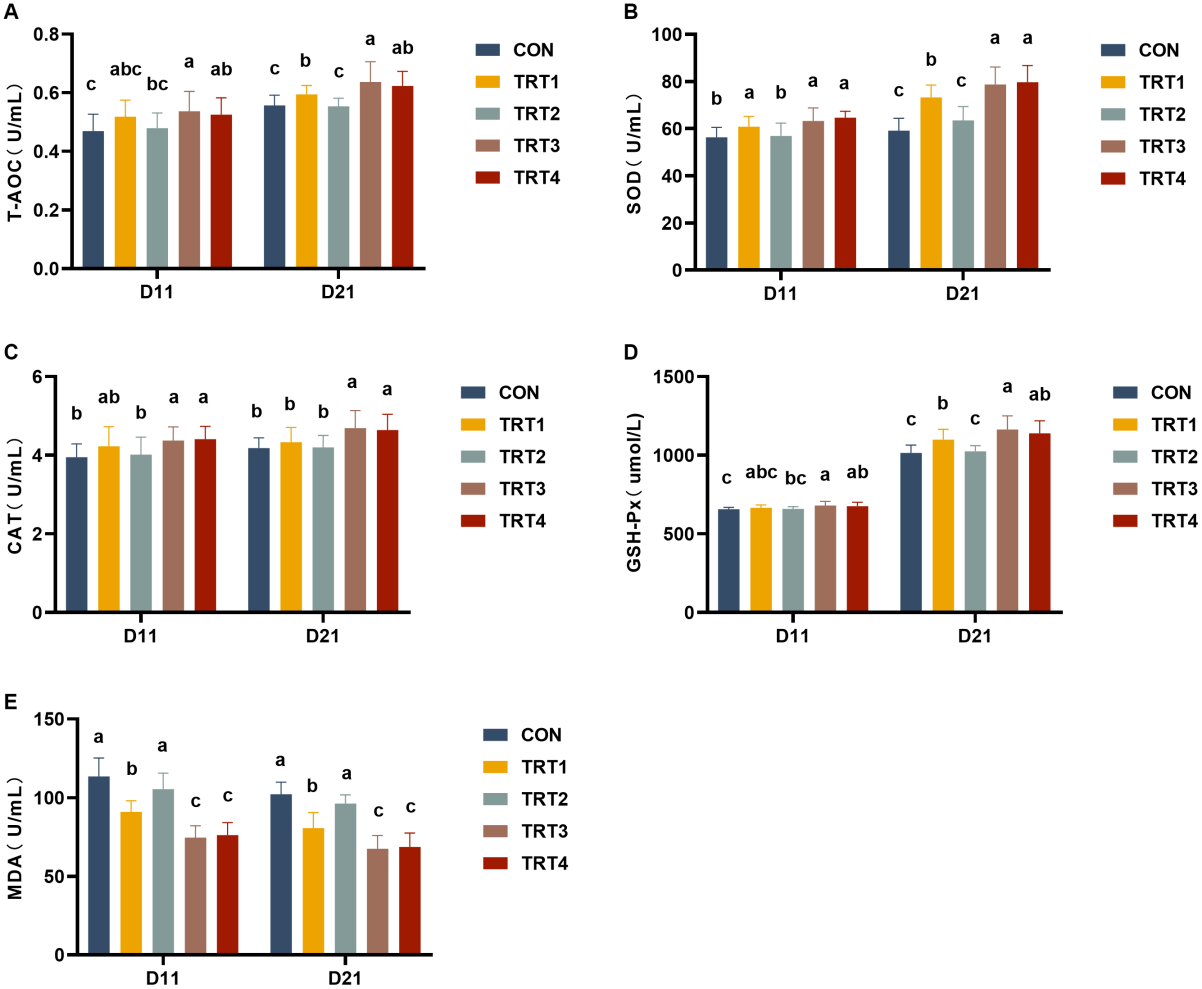
Effects of supplemental feeding of QZGSP to perinatal sows on serum antioxidant capacity in piglets. Concentrations of serum T-AOC **(A)**, SOD **(B)**, CAT **(C)**, GSH-Px **(D)**, and MDA **(E)** among the five groups from piglets on days 11 and 21 of lactation. Control (CON, basal diet), treatment group 1 (TRT1, basal diet +2 kg/t BZP), treatment group 2 (TRT2, basal diet +1 kg/t QZGSP), treatment group 3 (TRT3, basal diet +2 kg/t QZGSP), and treatment group 4 (TRT4, basal diet +3 kg/t QZGSP).

The results of serum antioxidant capacity in piglets are illustrated in Figure 2. As depicted in Figure 2A, piglets in the TRT3 and TRT4 groups exhibited significantly higher T-AOC contents compared to those in the CON group on day 11 of lactation (*p* < 0.05). Moreover, piglets in the TRT1, TRT3, and TRT4 groups demonstrated significantly higher concentrations of T-AOC than that observed in the CON group (*p* < 0.05), with a particularly notable increase observed in the T-AOC contents of the TRT3 group when compared to the TRT1 group on day 21 of lactation (*p* < 0.05). As shown in Figure 2B, sows within the TRT1, TRT3, and TRT4 groups displayed significantly increased concentrations of SOD relative to those within the CON group on days 11 and 21 of lactation (*p* < 0.05), and sows in TRT3 and TRT4 groups had significantly higher contents of SOD than those in TRT1 group on day 21 of lactation (*p* < 0.05). As depicted in Figure 2C, the concentrations of CAT in TRT3 and TRT4 groups were significantly higher than those in the CON group on days 11 and 21 of lactation (*p* < 0.05), the contents of CAT in TRT3 and TRT4 groups were significantly increased compared with TRT1 group on day 21 of lactation (*p* < 0.05). As shown in Figure 2D, piglets in the TRT1, TRT3, and TRT4 groups had significantly higher concentrations of GSH-Px than those in the CON group (*p* < 0.05), and Piglets in TRT3 group had significantly higher contents of GSH-Px than that observed in the TRT1 group on day 21 of lactation (*p* < 0.05). As shown in Figure 2E, compared with the CON group, piglets in TRT1, TRT3, and TRT4 groups had significantly lower contents of MDA than that in the CON group on days 11 and 21 of lactation (*p* < 0.05); compared with the TRT1 group, the concentrations of MDA in TRT3 and TRT4 groups were significantly reduced on days 11 and 21 of lactation (*p* < 0.05).

### 3.3 Effects of supplemental feeding of QZGSP to perinatal sows on gut microbiota sequence data of sows and their offspring piglets

To evaluate the effects of supplemental feeding of QZGSP to perinatal sows on gut microbiota diversity in sows and their offspring piglets, 16 s rRNA gene sequencing was performed using the fresh of sows and colon chyme of piglets. All 59 fresh fecal samples from sows were subjected to 16S rRNA gene sequencing (Supplementary Table S2), 3,425,898 raw tags and 2,271,918 raw tags were filtered to obtain 3,395,051 clean tags and 2,256,650 clean tags on day 0 and 21 of lactation, respectively. Additionally, all 30 colon chyme samples from piglets were subjected to 16S rRNA gene sequencing (Supplementary Table S2), 2,076,598 raw tags were filtered to collect 2,066, 280 clean tags on day 21 of lactation. These results indicated that the sequencing data met the criteria for further analysis.

**Figure 3:**
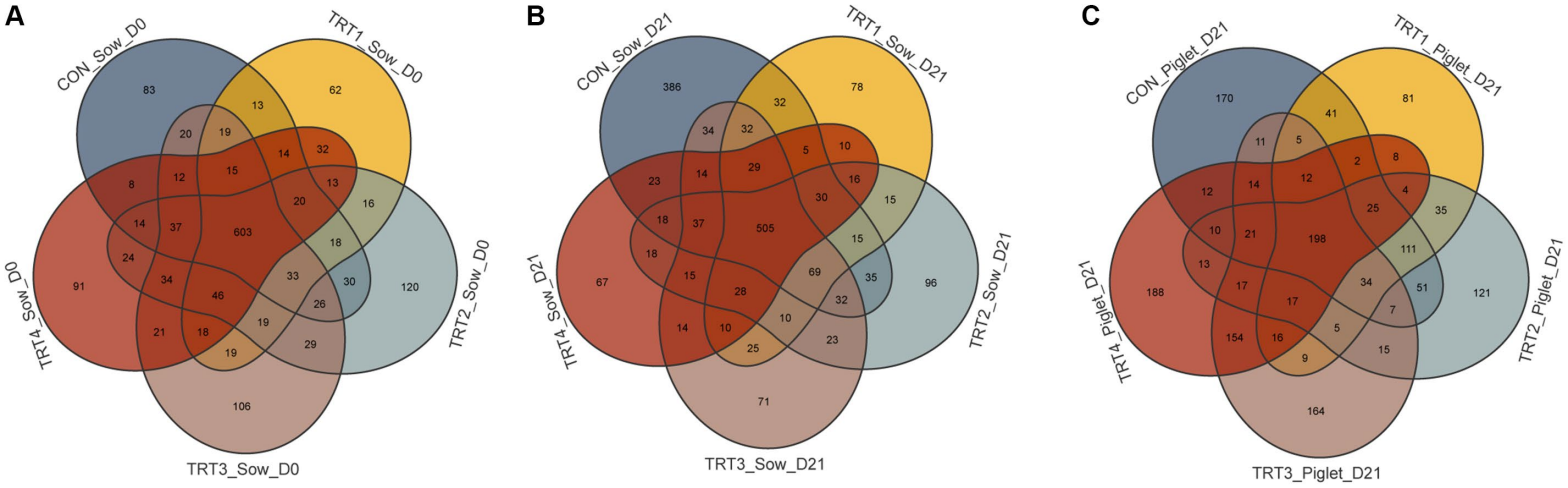
The Venn diagram for OTUs of the gut microbiota of sows and their offspring piglets among the five groups. **(A)** The Venn diagram for OTUs of gut microbiota in sows on day 0 of lactation. **(B)** The Venn diagram for OTUs of gut microbiota in sows on day 21 of lactation. **(C)** The Venn diagram for OTUs of gut microbiota in piglets on day 21 of lactation. Control (CON, basal diet), treatment group 1 (TRT1, basal diet +2 kg/t BZP), treatment group 2 (TRT2, basal diet +1 kg/t QZGSP), treatment group 3 (TRT3, basal diet +2 kg/t QZGSP), and treatment group 4 (TRT4, basal diet +3 kg/t QZGSP).

According to the statistics of the flattened OTUs (Figures 3A,B), there were 965, 960, 1,082, 1,090, and 1,001 OUTs from the fecal of sows in the CON, TRT1, TRT2, TRT3, and TRT4 groups on day 0 of lactation, respectively. Among them, there were 603 OUTs samples shared by all groups. The OTUs was higher in the TRT2, TRT3, and TRT4 groups, compared with the CON and TRT1 groups on day of lactation. We also collected 1,296, 909, 962, 948, and 839 OUTs from the fecal of sows in the CON, TRT1, TRT2, TRT3, and TRT4 groups on day 21 of lactation, respectively. Among them, there were 603 OUTs samples shared by all groups. The OTUs was lower in the TRT2, TRT3, and TRT4 groups, compared with the CON and TRT1 groups on day of lactation. In sum, dietary QZGSP supplementation during late gestation and lactation might better regulate the microorganism in the feces of sows. For the offspring piglets (Figure 3C), we obtained 724, 603, 684, 699, and 711 OTUs from the colon chyme of piglets in the CON, TRT1, TRT2, TRT3, and TRT4 groups on day 0 of lactation, respectively. Among them, there were 198 OUTs shared by all groups. The CON group had the highest number of OTUs of any group. Compared with the TRT1 group, the OTUs was higher in the TRT2, TRT3, and TRT4 groups on day 0 of lactation. In summary, dietary QZGSP supplementation could regulate the gut microbiota composition not only in sows, but also in their offspring piglets.

### 3.4 Effects of supplemental feeding of QZGSP to perinatal sows on gut microbiota diversity of sows and their offspring piglets

The effects of supplemental feeding of QZGSP to perinatal sows on the alpha diversity indices of gut microbiota in sows and their offspring piglets were presented in Supplementary Table S3. On day 0 of lactation, there were no significant differences in Chao1, Ace, Shannon and Simpson indices between groups of sows (*p* > 0.05). On day 21 of lactation, the control group exhibited the highest values of Chao1 and Ace index among all groups. The TRT2 and TRT3 groups demonstrated higher values of Chao1 and Ace index compared to the TRT1 group (*p* > 0.05), while the TRT4 group displayed lower values of Chao1 and Ace index than the TRT1 group (*p* > 0.05). Furthermore, on day 21 of lactation, the TRT3 group exhibited the lowest values for Shannon and Simpson’s indices among all groups, which were significantly lower than those in the CON group (*p* < 0.05) and comparable to those in the TRT1 group (*p* > 0.05). Conversely, the TRT4 group displayed higher values for Shannon and Simpson’s indices compared to the TRT1 group (*p* > 0.05).

**Figure 4:**
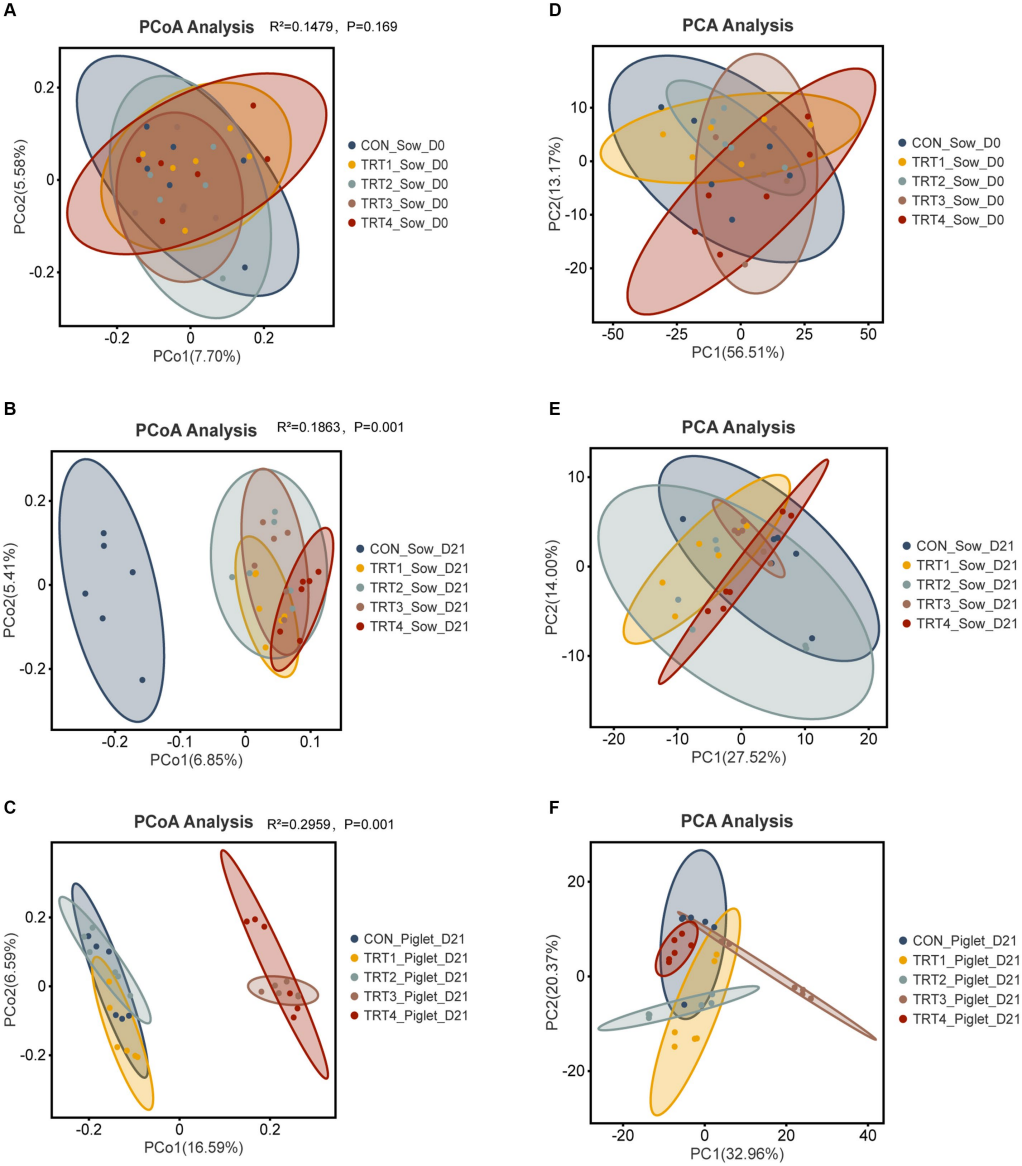
Effects of supplemental feeding of QZGSP to perinatal sows on beta-diversity of gut microbiota of sows and their offspring piglets. **(A)** PCoA for sows among the five groups on day 0 of lactation. **(B)** PCoA for sows among the five groups on day 21 of lactation. **(C)** PCoA for piglets among the five groups on day 21 of lactation. **(D)** PCA for sows among the five groups on day 0 of lactation. **(E)** PCA for sows among the five groups on day 21 of lactation. **(F)** PCA for piglets among the five groups on day 21 of lactation. Control (CON, basal diet), treatment group 1 (TRT1, basal diet +2 kg/t BZP), treatment group 2 (TRT2, basal diet +1 kg/t QZGSP), treatment group 3 (TRT3, basal diet +2 kg/t QZGSP), and treatment group 4 (TRT4, basal diet +3 kg/t QZGSP).

To investigate the impact of supplemental feeding of QZGSP to perinatal sows on the beta diversity of gut microbiota in sows and their offspring piglets, principal coordinate analysis (PCoA) was employed to assess group differences. PCoA was performed based on the unweighted_UniFrac distance metric, using OUT relative abundance data from the gut microbiota of both sows and their offspring piglets. The degree of correlation in the gut microbiota of sows during day 0 of the lactation period was relatively low, with principal component 1 (PC1) and PC2 explaining variations of 7.70 and 5.58%, respectively (Figure 4A). Similarly, during day 21 of the lactation period, the degree of correlation in the gut microbiota of sows remained low, with PC 1 and PC 2 explaining variations of 6.85 and 5.41%, respectively (Figure 4B). In piglets, during day 21 of their lactation period, there was a moderate level of correlation observed in their gut microbiota, with PC1 and PC2 explaining variations amounting to16.59 and 6.59%, respectively (Figure 4C). Subsequently, we employed the unweighted UniFrac distance algorithm to evaluate the statistical significance of alterations in the intestinal microbial community structure among sows and their offspring using the Adonis test (Supplementary material S4). From Figure 4A, we could know the sow’s fecal microbiota of TRT1 and TRT2 groups on day 0 of lactation were separated (Adonis: R^2^ = 0.1141, *p* = 0.045). As could be seen from Figure 4B, the sow’s fecal microbiota of CON group and TRT1 group on day 21 of lactation was separated (Adonis: R^2^ = 0.1417, *p* = 0.003). Similarly, CON and TRT2 groups (Adonis: R^2^ = 0.1372, *p* = 0.002), CON and TRT3 groups (Adonis: R^2^ = 0.1431, *p* = 0.001), and CON and TRT4 groups (Adonis: R^2^ = 0.1593, *p* = 0.005). Additionally, the sow’s fecal microbiota was separated in the TRT1 and TRT2 groups on day 21 of lactation (Adonis: R^2^ = 0.1054, *p* = 0.043), TRT1 and TRT3 groups (Adonis: R^2^ = 0.1115, *p* = 0.009), and TRT1 and TRT4 groups (Adonis: R^2^ = 0.1229, *p* = 0.003), respectively. According to Figure 4C, the piglet’s gut microbiota was separated in the CON and TRT1 groups (Adonis: R^2^ = 0.1351, *p* = 0.012), CON and TRT2 groups (Adonis: R^2^ = 0.1339, *p* = 0.001), CON and TRT3 groups (Adonis: R^2^ = 0.2606, *p* = 0.001), and CON and TRT4 groups (Adonis: R^2^ = 0.2377, *p* = 0.003) were distinctly segregated. Moreover, the piglet’s gut microbiota of TRT1 and TRT2 groups on day 21 of lactation were separated (Adonis: R^2^ = 0.1408, *p* = 0.002). Similarly, TRT1 and TRT3 groups (Adonis: R^2^ = 0.247, *p* = 0.002), TRT1 and TRT4 groups (Adonis: R^2^ = 0.2335, *p* = 0.002). We also assessed the similarity between the components of different sample groups using principal component analysis (PCA) (Supplementary material S4). The first and second principal components (PC1 and PC2) of intestinal microbiota in sows on day 0 of lactation were 7.70 and 5.58%, respectively (Figure 4D). On day 21 of lactation, the PC1 and PC2 values for intestinal microbiota in sows were determined as 27.52 and 14.00%, respectively (Figure 4E). For piglets at twenty-one days old, their intestinal microbiota had PC1 and PC2 values of 7.70 and 5.58%, respectively (Figure 4F).

### 3.5 Effects of supplemental feeding of QZGSP to perinatal sows on gut microbiota composition in sows and their offspring piglets

**Figure 5:**
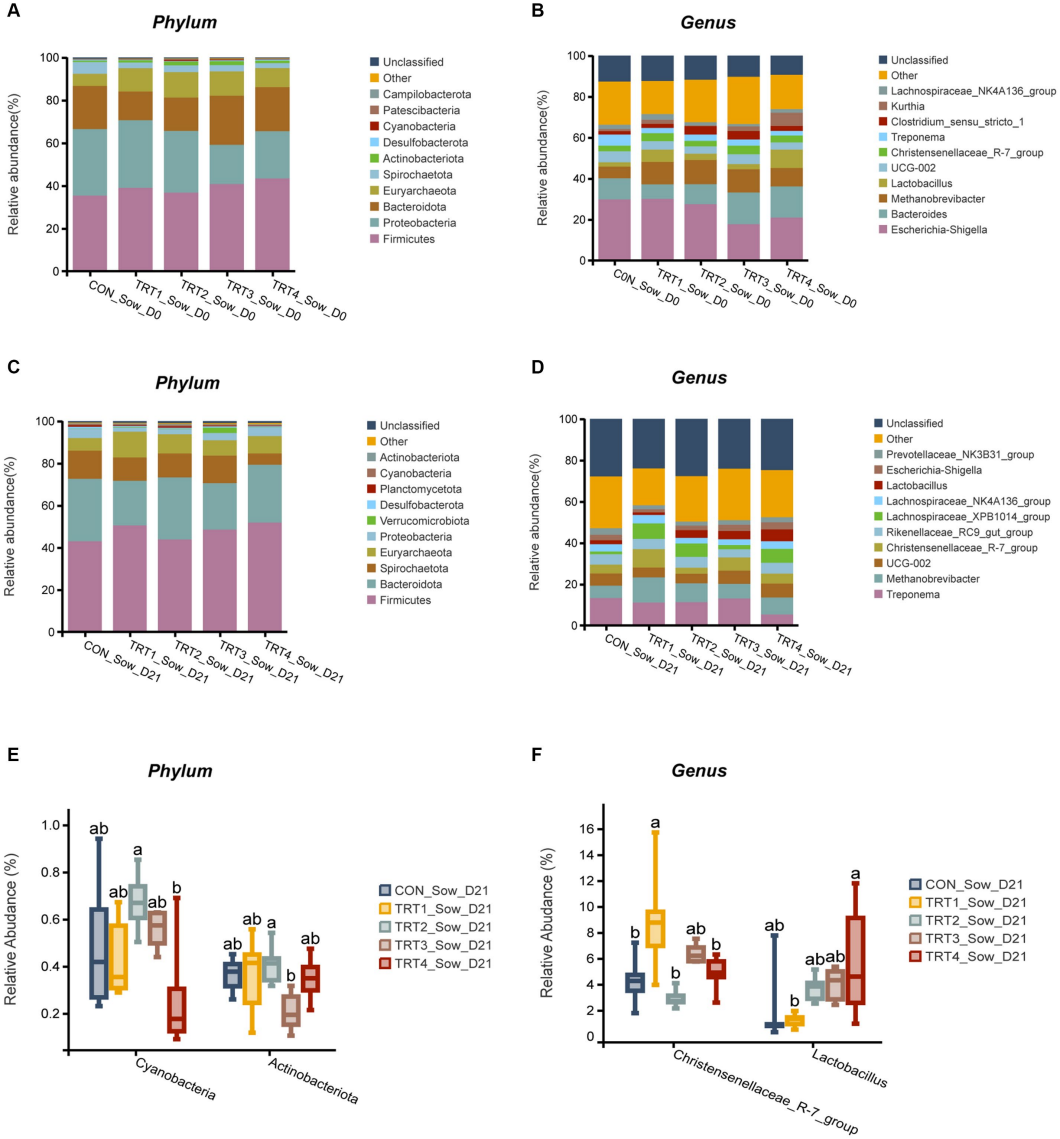
Effects of supplemental feeding of QZGSP to perinatal sows on gut microbiota composition of sows. **(A)** Relative abundance at the phylum level of sows on day 0 of lactation. **(B)** Relative abundance at the genus level of sows on day 0 of lactation. **(C)** Relative abundance at the phylum level of sows on day 21 of lactation. **(D)** Relative abundance at the genus level of sows on day 21 of lactation. **(E)** Significance test at the phylum level of sows on day 21 of lactation. **(F)** Significance test at the genus level of sows on day 21 of lactation. Control (CON, basal diet), treatment group 1 (TRT1, basal diet +2 kg/t BZP), treatment group 2 (TRT2, basal diet +1 kg/t QZGSP), treatment group 3 (TRT3, basal diet +2 kg/t QZGSP), and treatment group 4 (TRT4, basal diet +3 kg/t QZGSP).

The effects of supplemental feeding of QZGSP to perinatal sows on gut microbiota composition of sows on day 0 of lactation were presented in Figures 5A,B. At the phylum level (Figure 5A), the abundance of Proteobacteria was lower in the TRT3 and TRT4 groups, compared with sows in the CON and TRT1 groups, the abundance of Bacteroidota was greater in the TRT3 and TRT4 groups than that in the TRT1 group. At the genus level (Figure 5B), the abundance of *Eschericha-Shigella* was lower in the TRT3 and TRT4 groups, compared with the CON and TRT2 groups (Figure 5B), the abundance of Bacteroides was greater in TRT3 and TRT4 groups than in the CON and TRT1 group, the abundance of *Lactobacillus* and *Kurthia* was higher in the TRT4 group, compared with the CON and TRT1 groups.

The effects of supplemental feeding of QZGSP to perinatal sows on gut microbiota composition of sows on day 21 of lactation were presented in Figures 5C–F. At the phylum level, the abundance of *Bacteroidota* was higher than that observed in the TRT2 group (Figure 5C). At the genus level, the abundance of Treponema was lower in the TRT4 group, compared with the CON and TRT1 groups (Figure 5D). As shown, at the genus level (Figure 5F), the abundance of *Christensenellaceae_R-7_group* in TRT1 groups was significantly higher than that in the CON, TRT3, and TRT4 groups (*p* < 0.05). Compared with the CON group, the abundance of *Lactobacillus* in the TRT4 group was significantly increased (*p* < 0.05).

**Figure 6:**
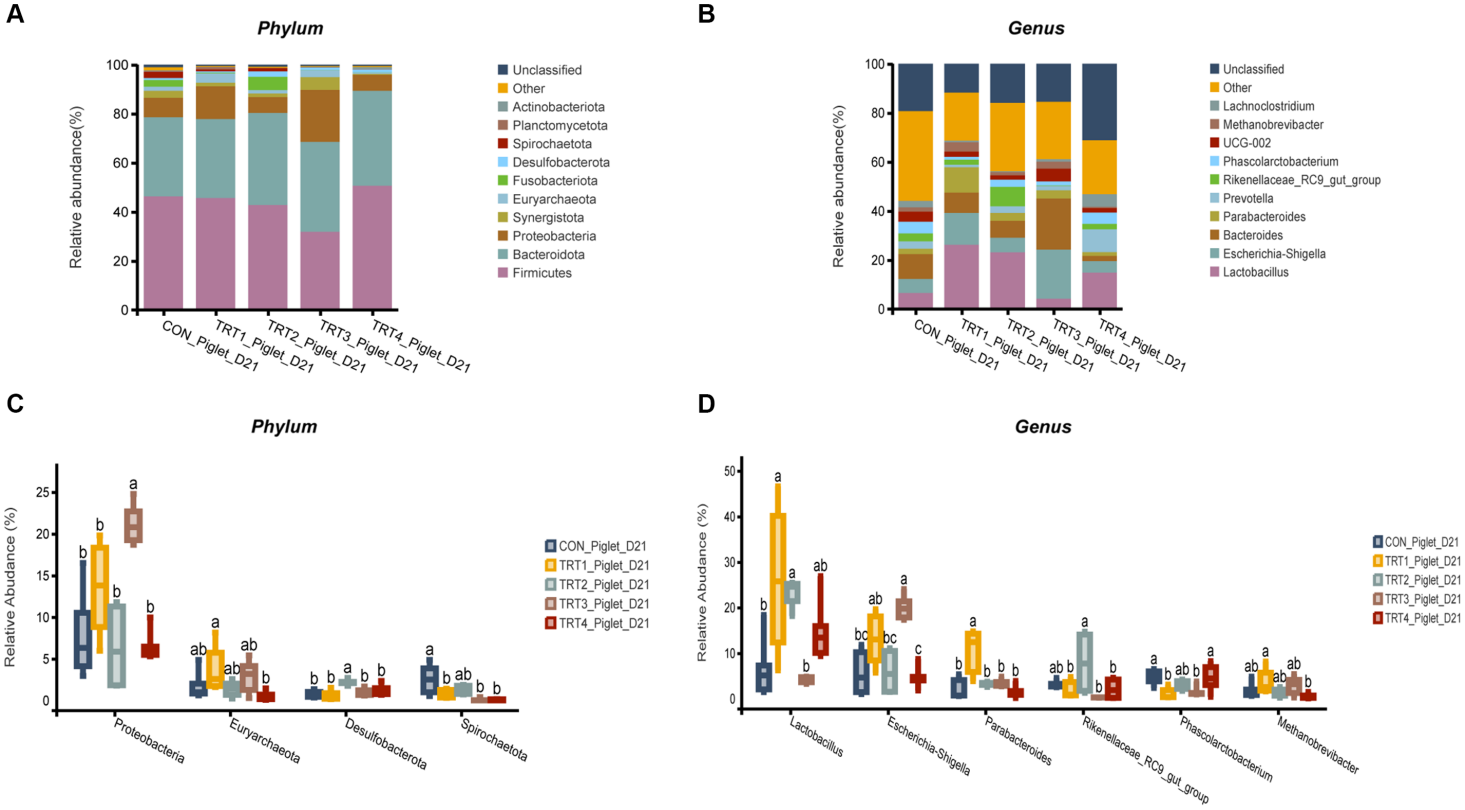
Effects of supplemental feeding of QZGSP to perinatal sows on gut microbiota composition of piglets. **(A)** Relative abundance at the phylum level of piglets on day 21 of lactation. **(B)** Relative abundance at the genus level of piglets on day 21 of lactation. **(C)** Significance test at the phylum level of piglets on day 21 of lactation. **(D)** Significance test at the genus level of piglets on day 21 of lactation. Control (CON, basal diet), treatment group 1 (TRT1, basal diet +2 kg/t BZP), treatment group 2 (TRT2, basal diet +1 kg/t QZGSP), treatment group 3 (TRT3, basal diet +2 kg/t QZGSP), and treatment group 4 (TRT4, basal diet +3 kg/t QZGSP).

The effects of supplemental feeding of QZGSP to perinatal sows on intestinal microbiological composition of offspring piglets on day 21 of lactation were shown in Figure 6. At the phylum level (Figures 6A,C), the abundance of Firmicutes in the TRT4 group was greater higher than that observed in the TRT1 and CON groups, the abundance of *Proteobacteria* in the TRT3 group was higher than those in TRT1 and CON groups. Besides, compared with the CON and TRT1 groups, the abundance of *Proteobacteria* in the TRT3 group was significantly increased (*p* < 0.05). The abundance of *Euryarchaeota* in the TRT4 group was significantly higher than that in the TRT1 group (*p* < 0.05). The abundance of *Desulfobacterota* a in the TRT3 group was significantly higher than that in the CON and TRT1 groups (*p* < 0.05). The abundance of *Spirochaetota* in the CON group was significantly lower than that in TRT1, TRT3, and TRT4 groups (*p* < 0.05). At the genus level (Figures 6B,D), the abundance of *Bacteroides* in the TRT3 group was higher than that in the CON and TRT1 groups. Besides, the abundance of *lactobacillus* in the TRT1 and TRT2 groups was significantly higher than that observed in the CON group (*p* < 0.05), the abundance of *lactobacillus* in the TRT3 group was significantly lower than those in the TRT1 group (*p* < 0.05). The abundance of *Escherichia-Shigella* in the TRT4 group was significantly than that observed in the TRT1 group (*p* < 0.05). The abundance *Parabacteroides* in the TRT1 groups was significantly higher than those in the CON group (*p* < 0.05), compared with TRT1 group, the abundance of *Parabacteroides* in the TRT2, TRT3, and TRT4 groups was significantly reduced (*p* < 0.05). The abundance of *Phascolarctobacterium* of TRT1 and TRT2 groups was significantly higher than in the CON group (*p* < 0.05), the abundance of *Phascolarctobacterium* in the TRT4 groups was significantly higher than those in the TRT1 group (*p* < 0.05). Compared with the TRT1 group, the abundance of *Methanobrevivacter* in the TRT4 group was significantly increased (*p* < 0.05).

**Figure 7:**
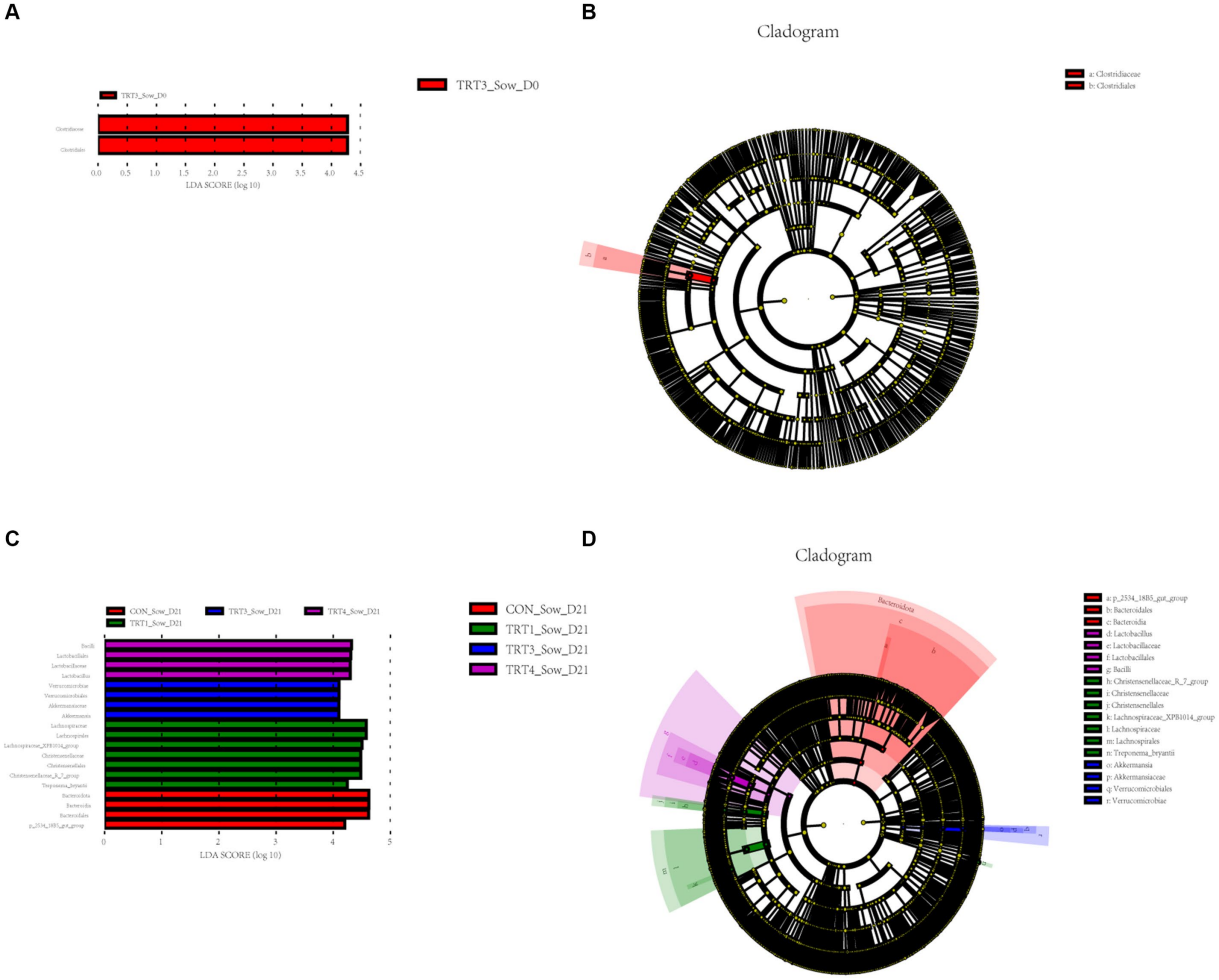
Linear discriminant analysis (LDA) effect size (LEfSe) analysis of the gut microbiota in sows. The LEfSe analysis histogram identified the differentially abundant (LDA score > 4) bacterial taxa among different groups on days 0 **(A)** and 21 **(C)** of lactation. The ordinate represents the taxa exhibiting statistically significant differences between groups, while the abscissa displays a bar graph depicting the LDA logarithmic score value for each taxon. The length of the bar indicates the magnitude of difference for each taxon, with longer bars indicating greater significance. Additionally, the color of each bar corresponds to the sample group with the highest abundance for that particular taxon. The LEfSe analysis cladogram showing the most discriminative bacterial clades identified by LEfSe among different groups on days 0 **(B)** and 21 **(D)** of lactation. The size of each node corresponds to the average relative abundance of the taxa, while hollow nodes indicate taxa with statistically insignificant differences between groups. The letters serve as identifiers for taxa that exhibit significant differences between the groups. Control (CON, basal diet), treatment group 1 (TRT1, basal diet +2 kg/t BZP), treatment group 2 (TRT2, basal diet +1 kg/t QZGSP), treatment group 3 (TRT3, basal diet +2 kg/t QZGSP), and treatment group 4 (TRT4, basal diet +3 kg/t QZGSP).

**Figure 8:**
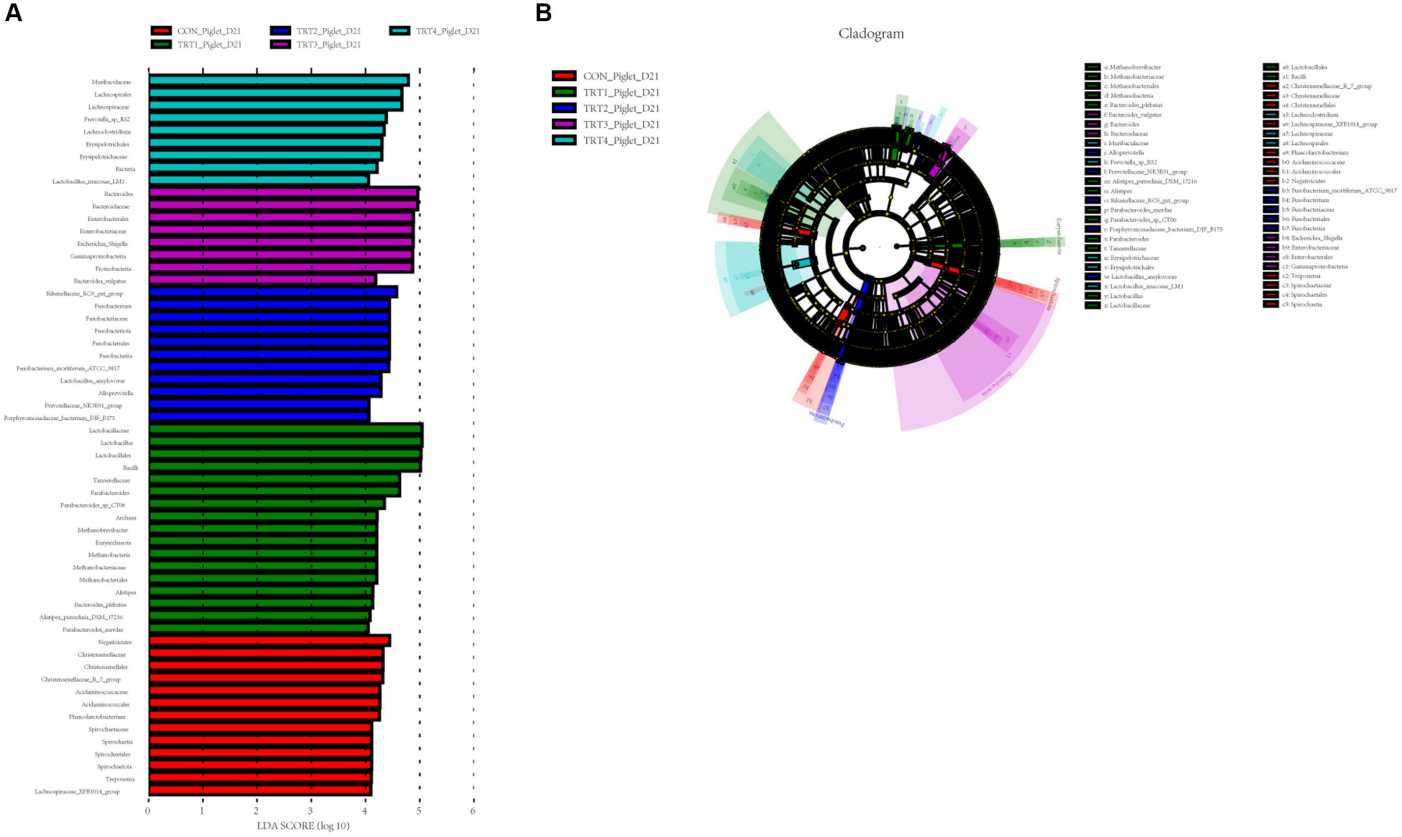
Linear discriminant analysis (LDA) effect size (LEfSe) analysis of the gut microbiota in piglets. The LEfSe analysis histogram identified the differentially abundant (LDA score > 4) bacterial taxa among different groups on days 21 **(A)** of lactation. The ordinate represents the taxa exhibiting statistically significant differences between groups, while the abscissa displays a bar graph depicting the LDA logarithmic score value for each taxon. The length of the bar indicates the magnitude of difference for each taxon, with longer bars indicating greater significance. Additionally, the color of each bar corresponds to the sample group with the highest abundance for that particular taxon. The LEfSe analysis cladogram showing the most discriminative bacterial clades identified by LEfSe among different groups on day 21 **(B)** of lactation. The size of each node corresponds to the average relative abundance of the taxa, while hollow nodes indicate taxa with statistically insignificant differences between groups. The letters serve as identifiers for taxa that exhibit significant differences between the groups. Control (CON, basal diet), treatment group 1 (TRT1, basal diet +2 kg/t BZP), treatment group 2 (TRT2, basal diet +1 kg/t QZGSP), treatment group 3 (TRT3, basal diet +2 kg/t QZGSP), and treatment group 4 (TRT4, basal diet +3 kg/t QZGSP).

Next, to identify bacterial taxa that significantly differentiated in fecal of sows among groups, a linear discriminant analysis effect size (LEfse) analysis (LDA score > 4) was performed (Figure 7). The cladogram reports the taxa showing different abundant values according to LefSe (Figure 8). On day 0 of lactation (Figure 7A), *Clostridiaceae* (family) and *Clostridiales*(order)were more abundant in the TRT3 group. On day 0 of lactation (Figure 7B), only 2 different taxonomic levels of microorganisms were identified as potential biomarkers in the TRT3 group. On day 21 of lactation (Figure 7C), *Bacilli* (class), *Lactobacillales* (order), *Lactobacillaceae* (family), and *Lactobacillus* (genus) was more enriched in TRT4 group; *Verrucomicrobiae* (class), *Verrucomicrobiales* (order), *Akkermansiaceae* (family), and *Akkermansia* (genus) were more abundant in TRT3 group; *Lachnospiraceae* (family), *Lachnospirales* (order), *Lachnospiraceae_XPB1014_group* (genus), *Christensenellaceae* (family), *Christensenellales* (order), *Christensenellaceae_R_7_group* (genus), and *Treponema_bryantii* (species) were more enriched in the TRT1 group. *Bacteroidota* (phylum), *Bacteroidia* (class), *Bacteroidales* (order), and *p_2534_18B5_gut_group* (genus) was more abundant in the CON group. On day 21 of lactation (Figure 7D), there were 3, 7, 4, and 4 different taxonomic levels of microorganisms were identified as potential biomarkers in the CON, TRT1, TRT3, and TRT4 groups, respectively.

For the offspring piglets, to identify taxa that significantly differentiated in colon chyme of piglets among groups, a linear discriminant analysis effect size (LEfse) analysis (LDA > 4) was performed (Figure 8). On day 21 of lactation (Figure 8A), *Muribaculaceae* (phylum), *Lachnospirales* (order), *Lachnospiraceae* (family), *Prevotella_sp_RS2* (species), *Lachnoclostridium* (genus), *Erysipelotrichales* (order), *Erysipelotrichaceae* (family), *Bacteria*, and *Lactobacillus_mucosae_LM1*(family) were more abundant in the TRT4 group; *Bacteroides* (genus), *Bacteroidaceae* (phylum), *Enterobacterales* (order), *Enterobacteriaceae* (family), *Escherichia_Shigella* (genus), *Gammaproteobacteria* (class), *Proteobacteria* (phylum), and *Bacteroides_vulgatus* were more enriched in the TRT3 group; *Rikenellaceae_RC9_gut_group* (genus), *Fusobacterium* (genus), *Fusobacteriaceae* (phylum), *Fusobacteriota* (phylum), *Fusobacteriales* (order), *Fusobacteria* (phylum), *Fusobacterium_mortiferum_ATCC_9817* (domian), *Lactobacillus_amylovorus* (genus), *Alloprevotella* (genus), *Prevotellaceae_NK3B31_group* (genus), and *Porphyromonadaceae_bacterium_DJF_B175* (family) were more abundant in the TRT2 group; *Lactobacillaceae* (phylum), *Lactobacillus* (genus), *Bacilli* (class), *Lactobacillales* (order), *Tannerellaceae* (family), *Parabacteroides* (genus), *Parabacteroides_sp_CT06* (genus), *Archaea*, Methanobrevibacter (genus), *Euryarchaeota* (phylum), *Methanobacteria* (class), *Methanobacteriaceae* (family), *Methanobacteriales* (order), *Alistipes* (genus), *Bacteroides_plebeius* (genus), *Alistipes_putredinis_DSM_17216* (genus), and *Parabacteroides_merdae* (genus) were more enriched in the TRT1 group; *Negativicutes* (class), *Christensenellaceae* (family), *Christensenellales* (order), *Christensenellaceae_R_7_group* (phylum), *Acidaminococcaceae* (family), *Acidaminococcales* (genus), *Phascolarctobacterium* (genus), *Spirochaetaceae* (phylum), Spirochaetia (class), *Spirochaetales* (order), *Spirochaetota* (phylum), *Treponema* (genus), *Lachnospiraceae_XPB1014_group* (family) were more enriched in the CON group. The cladogram reports the taxa showing different abundant values according to LefSe. On day 21 of lactation (Figure 8B), there were 12, 15, 10, 7, and 7 different taxonomic levels of microorganisms were identified as potential biomarkers in the CON, TRT1, TRT2, TRT3, and TRT4 groups, respectively.

### 3.6 Correlation analysis between the differential gut microbiota and serum antioxidant capacity

**Figure 9:**
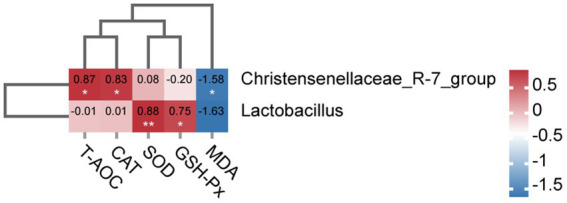
Heatmap of the correction analysis between the differential gut microbiota and serum antioxidant capacity of sows on day 21 of lactation. The red color indicates a positive correlation, while the blue color signifies a negative correlation (* *p* < 0.05; ** *p* < 0.01).

To further investigate the association between gut microbiota and oxidative stress in sows on day 21 of lactation, we conducted the association analysis between the differential microbiota at the genus level of the top 10 abundant genera and serum antioxidant capacity. As shown in Figure 9, the Spearman correlation matrix illustrated that the relative abundance of *Christensenellaceae_R-7_group* was positively correlated with the levels of serum T-AOC and CAT (*p* < 0.05), but negatively correlated with the concentration of serum MDA (*p* < 0.05). Meanwhile, the relative abundance of *Lactobacillus* was positively correlated with the concentration of serum SOD (*p* < 0.01) and GSH-Px (*p* < 0.05).

## 4 Discussion

Chinese herbal medicine (CHM) and their extracts are extensively as health promoters and innovative approaches for disease treatment (Li and Weng, 2017). Chinese herbal medicine (CHM) contains a wide range of bioactive compounds that offer various nutritional and health benefits to animals, making them commonly utilized as feed additives in livestock (Abdallah et al., 2019). Unfortunately, scientific studies on the effects of supplemental feeding of Chinese herbal mixture (CHM) to perinatal sows on the antioxidant capacity and gut microbiota of sows and their offspring piglets are limited. In this study, we observed that the inclusion of 3 kg/t QGZSP in sow diets during late gestation and lactation resulted in increased antioxidant capacity in both sows and their offspring piglets, as well as modulation of the gut microbiota composition in both groups. Moreover, the overall outcomes were found to be superior compared to those achieved with 2 kg/t BZP, highlighting the potential benefits of incorporating QZGSP as a feed additive for sows during late gestation and lactation.

It is widely acknowledged that high-yielding sows experience heightened oxidative stress during late gestation and lactation, which in turn affects the lactation performance, farrowing performance, and immunity of sows (Ao and Peng, 2016). Due to the naive antioxidant system, newborn piglets are unable to efficiently eliminate excessive free radicals and consequently experience oxidative stress (Yin et al., 2013). The premature separation of piglets from their sows, coupled with the stress induced by dietary and environmental changes, in conjunction with the incomplete development of the piglet’s organ systems, leads to a decrease in feed intake and disruptions in both immune system function and intestinal health (Zheng et al., 2021). The contents of total antioxidant capacity (T-AOC), Superoxide Dismutase (SOD), Glutathione Peroxidase (GSH-PX), Catalase (CAT), were currently employed to evaluate the antioxidant stress of pigs (Pardo et al., 2021). The concentration of MDA serves as an indicator for assessing the degree of lipid peroxidation and compromised antioxidative capacity in animals (Bednarz-Misa et al., 2020). Next, we explored the effects of supplemental feeding of QZGSP to perinatal sows on the serum antioxidant capacity of sows and their offspring piglets. In the present study, compared to the CON and TRT1 groups, the serum concentration of T-AOC, SOD, GSH-Px, and CAT in the TRT3 or/and TRT4 groups from sows and their offspring piglets exhibited a significant increase. Conversely, the contents of MDA in the serum of sows and their offspring piglets were significantly reduced in both the TRT3 and TRT4 groups. The maintenance of animal health relies heavily on the organism’s antioxidant status, which can be influenced by dietary nutrition (Huo et al., 2020; Liu et al., 2023). Given the inherent antioxidant properties found in herbs, incorporating them as additives becomes crucial for bolstering the animals’ antioxidant system and enhancing stress tolerance. Single herbs or combinations of herbal extracts with antioxidant capacity have been widely employed in livestock industry (Liang et al., 2013; Xinhong et al., 2022; Liu et al., 2024). For instance, a previous study has indicated that the administration of *A. polysaccharide* and *A. membranaceus* root can significantly improve the overall antioxidant capacity and total superoxide dismutase activities in lambs (Zhong et al., 2012). Similarly, Wu et al. (2021) reported that a mixture of soybean isoflavone and *astragalus polysaccharide* could augment the serum antioxidant activity in lactating sows (Wu et al., 2021). Paeoniflorin effectively attenuated cell apoptosis, increased cell viability, and suppressed reactive oxygen species (ROS) accumulation by upregulating the expressions of superoxide dismutase (SOD) and antioxidant enzymes catalase (CAT), which are antioxidant enzymes (Yuan et al., 2020). Pan et al. (2024) reported that supplementation *B. Radix* in the diet of weaning Shanbei fine-wool sheep could enhance antioxidant capacity while reducing oxidant stress levels. Long et al. (2023) reported that *A. sinensis* extract elevated the levels of antioxidant enzymes in copper-exposed fish serum, thereby mitigating oxidative damage induced by copper exposure. The results demonstrated that the supplementation of QZGSP in perinatal sows diets significantly enhanced the antioxidant capacity of sows and their offspring piglets, thereby effectively mitigating oxidative stress-induced effects. This phenomenon can be attributed to the presence of a diverse of bioactive constituents with potent antioxidant properties within the six Chinese herbs used in QZGSP.

The gut microbiota is an intricate and varied ecology made up of billions of microorganisms that are vital to host immunity, metabolism, and behavior (Huang and Chen, 2023). When investigating the human gut microbiota for immunomodulatory organisms, researchers have discovered that microbial diversity plays a crucial role in ensuring consistent immune regulation ability. Therefore, comprehending microbial diversity becomes paramount (Geva-Zatorsky et al., 2017). Periparturient sows exhibited susceptibility to the metabolic syndrome, leading to a decline in the diversity of the intestinal microbiota (Cheng et al., 2018). The Chao1, Ace, Shannon and Simpson indices are commonly utilized for evaluating microbiota diversity. The study results revealed that the control group exhibited the highest Chao1 and Ace index values on day 21 of lactation, while the TRT2 and TRT3 groups demonstrated higher Chao1 and Ace indexes compared to the TRT1 group on day 21 of lactation (*p* > 0.05). Conversely, the Chao1 and Ace indexes were lower in the TRT4 group than in the TRT1 group on day 21 of lactation (*p* > 0.05). These findings suggest that QZGSP enhances bacterial community diversity, with changes potentially influenced by drug dosage and duration. However, the TRT3 group exhibited reduced levels of intestinal microflora diversity from piglets on day 21 of lactation compared to the control group. Weaning stress induces dysbiosis and translocation of the intestinal microflora in piglets (Tang et al., 2022). Previous studies have reported that the increase in intestinal flora diversity and richness of piglets mainly occurred before the 21st day of birth, and stabilized or slightly decreased after weaning (Frese et al., 2015; Arfken et al., 2020). In our study, the TRT3 group exhibited significantly reduced levels of intestinal microflora diversity on day 21 of lactation compared to the blank control group (*p* > 0.05). Meanwhile, there was no significant disparity in the diversity of intestinal flora between the QZGSP-supplemented and BZP-supplemented sow diets. This phenomenon may be attributed to the inhibitory effect of Chinese herbal mixture (CHM) on the colonization of pathogenic bacteria in the intestine, leading to a reduction in intestinal flora diversity. The beta diversity analysis results indicated that there was no statistically significant difference between the CON group and the other treatment groups from sows based on the PCoA during day 0 of the sow’s lactation period. However, a significant difference was observed between the CON group and the other treatment groups based on the PCoA during day 21 of the sow’s lactation period. Furthermore, a significant distinction was found between the TRT1 group and the TRT2, TRT3, and TRT4 groups based on the PCoA at 21 days of age in piglets. These findings indicated that the influential effects of QZGSP on the taxonomic structure of the intestinal microbiota gradually became enhance. These results indicate that the effect of QZGSP in the diet of sows on the taxonomic structure of the intestinal microbiota of sows gradually increased and was more pronounced on the taxonomic structure of the intestinal microbiota of the offspring piglets at 21 days of lactation.

In addition to its role in microbial diversity, our study also indicated that the addition of QZGSP to perinatal sows’ diet during late gestation and lactation had an impact on the abundance of intestinal flora in sows and their offspring piglets. In this experiment, the dominant phyla in the intestinal microbiome of sows and their offspring piglets were *Firmicutes* and *Bacteroidetes*, which is in general agreement with previous reports (Isaacson and Kim, 2012; Wang R. et al., 2021). Our research further revealed that dietary inclusion of the QZGSP in sow diets resulted in a notable shift in gut microbiota composition. Specifically, at the phylum and genus levels, the relative abundance of *Proteobacteria* and *Escherichia-Shigella* in the TRT3 and TRT4 groups from sows was less than other groups on day 0 of lactation. However, unexpectedly, the relative abundance of the *Escherichia-Shigella* increased in the piglets of TRT3 group on day 21 of lactation was higher than the CON group on day 21 of lactation, while the relative abundance of *Escherichia-Shigella* decreased in the piglets of TRT4 group was significantly lower than the TRT1 group on day 21 of lactation. The current understanding suggests that *Escherichia-Shigella* is a prevalent pathogen associated with diarrhea, and an elevated population of Shigella may contribute to the development of intestinal inflammation (Sousa et al., 2010). Yang et al. (2024) reported that cholestasis disrupts the structure and composition of the gut microbiota, leading to an increase in the abundance of Escherichia-Shigella enriched in virulence factor. Our speculation is that this could be associated with the quantity of bile acids that have been introduced. The microorganism *Christensenellaceae_R.7_Group* is widely distributed in both humans and animals, and it has been primarily associated with obesity and inflammatory bowel disease (Waters and Ley, 2019). Previous study has reported a relatively low relative abundance of the *Christensenellaceae_R.7_group* in obese patients (Valdes et al., 2018). However, it is noteworthy that obese individuals generally exhibit an increased risk of endometritis compared to the general population (Kalkanbaeva et al., 2017). Our study also found that the relative abundance of Christensenellaceae_R-7_ group from sows were significantly lower in the TRT2 and TRT4 groups compared to the TRT1 group on the 21st day of lactation, indicating that supplemental feeding of QZGSP to perinatal sows may effectively reduce the incidence of endometritis in sows. The *Lactobacillus* strain is a type of beneficial bacteria that plays a crucial role in maintaining the balance of intestinal microflora and enhancing human immune function (Roujie et al., 2022). In the present study, at the genus level, the relative abundance of *Lactobacillus* in the TRT4 group form sows was greater than those in the CON group on the day 21 of lactation. Therefore, we concluded that supplementation of 3 kg/t QZGSP in the diets of perinatal sows increases the relative abundance of Lactobacillus and thus promoting sow health. However, at the genus level, the abundance of *Lactobacillus* in the TRT4 group form sows was significantly lower in the TRT2 and TRT4 groups compared to the TRT1 group on the 21st day of lactation. We hypothesize that supplementation of QZGSP in sow feed during late gestation and lactation may enhance lactic acid production in the intestines of their offspring piglets, thereby suppressing Lactobacillus proliferation. As was previously mentioned, during the fermentation process, lactic acid prevents Lactobacillus from proliferating (Chen et al., 2021). As previous reported, the abundance of *Parabacteroides* has been found to increase in human diseases such as alopecia areata (Moreno-Arrones et al., 2020), gestational diabetes mellitus (Kuang et al., 2017), polycystic ovary syndrome (Chu et al., 2020), and nonalcoholic steatohepatitis (Wong et al., 2013). In the present study, at the genus level, we found that the abundance of Parabacteroides was significantly lower in the TRT2, TRT3 and TRT4 groups than in the TRT1 group on day 21 of lactation, suggesting that the addition of QZGSP to the diets of perinatal sows may improve herd health and avoid the occurrence of diseases similar to those mentioned above, but the specific mechanism of action needs to be further investigated.

The correlation analysis between two variables is often confounded by the presence of other variables when multiple factors are involved (Luo et al., 2010). Spearman correlation matrix illustrated that the relative abundance of *Christensenellaceae_R-7_group* was positively associated with the CAT and T-AOC concentrations in the sow serum, and was negatively associated with the MDA on day 21 of lactation. Our study also found that the abundance of *Lactobacillus* was positively associated with the GSH-Px and SOD contents in the sow serum on day 21 of lactation. The antioxidant effect of Lactobacillus has been demonstrated, along with its ability to mitigate oxidative stress injury by reducing levels of reactive oxygen species (ROS) (Kong et al., 2020). These findings suggest that incorporating Chinese herbal mixture (CHM) into the diets of perinatal sows during late gestation and lactation could optimize the abundance of Christensenellaceae_R-7_group and Lactobacillus in the gut, thereby enhancing antioxidant capacity and mitigating oxidative damage in both sows and their offspring piglets.

## 5 Conclusion

In summary, the supplementation of 3 kg/t QZGSP in periparturient sow diet effectively improved antioxidant capacity in sows and their offspring piglets, modulated the composition and structure of gut microbiota in sows and their offspring piglets. QZGSP has the potential to be developed as a feed additive for periparturient sows.

## Data Availability

The datasets presented in this study can be found in online repositories. The names of the repository/repositories and accession number(s) can be found below: NCBI, PRJNA1131757.

## Footnotes

^1^ http://www.omicsmart.com
