# Supplementary material for: Effects of supplemental feeding of Chinese herbal mixtures to perinatal sows on antioxidant capacity and gut microbiota of sows and their offspring piglets
Source: Front Microbiol. 2024 Sep 12;15:1459188. doi: 10.3389/fmicb.2024.1459188 (PMC11424466; doi:10.3389/fmicb.2024.1459188)
Supplement: Supplementary file 1 [file Data_Sheet_1.ZIP › Supplementary files/Table 1.DOCX]

Supplementary Material

# Supplementary Table 1. Composition and nutrient levels of the basal diet (as fed basis).

| **Items** | **Late gestation** | **Lactation** |
| --- | --- | --- |
| Ingredients (%) |  |  |
| Corn | 50.04 | 47.65 |
| Barley | 17.4 | 18 |
| Soybean meal | 17.2 | 19 |
| Expanded soybean | 6 | 6 |
| Fish meal | 2 | 2 |
| NaCl | 0.4 | 0.4 |
| CaHPO_4_ | 1.4 | 1.4 |
| Limestone | 1.6 | 1.6 |
| Lys | 0.26 | 0.25 |
| Soybean oil | 2.7 | 2.7 |
| Premix^1^ | 1 | 1 |
| Total | 100.00 | 100.00 |
| Nutrient levels (%) |  |  |
| DM (MJ/kg) | 14.20 | 14.30 |
| CP | 15.40 | 15.90 |
| EE | 5.00 | 5.10 |
| Ash | 5.80 | 5.90 |
| CF | 3.90 | 3.50 |
| Ca | 1.07 | 1.20 |
| P | 0.50 | 0.59 |
| AP | 0.40 | 0.45 |
| Lys | 1.14 | 1.17 |
| Met | 1.10 | 0.99 |

Note: ^1^Premix during pregnancy: Cu 5 mg, I 0.15 mg, Fe 83mg, Mn 20 mg, Zn 128 mg, VA 13400 IU, VD_3_ 2800 IU, Choline chloride 1000 mg, VE 22.4 mg, VK_3_ 3 mg. Lactation premix: Cu 15 mg, Fe 82 mg, I 0.13 mg, Mn 20 mg, Zn 128 mg, VA 10000 IU, VD_3_ 2000 IU, VK_3_ 1.5 mg, VE 30 mg. ^2^ The DE was calculated and others were measured.
