# Supplementary material for: Effects of supplemental feeding of Chinese herbal mixtures to perinatal sows on antioxidant capacity and gut microbiota of sows and their offspring piglets
Source: Front Microbiol. 2024 Sep 12;15:1459188. doi: 10.3389/fmicb.2024.1459188 (PMC11424466; doi:10.3389/fmicb.2024.1459188)
Supplement: Supplementary file 1 [file Data_Sheet_1.ZIP › Supplementary files/Table 3.DOCX]

Supplementary Material

# Supplementary Table 3. Effect of dietary QZGSP supplementation on the alpha diversity of gut microbiota in sows and offspring piglets

| **Items** | | **CON** | **TRT1** | **TRT2** | **TRT3** | **TRT4** | ***P*-value** |
| --- | --- | --- | --- | --- | --- | --- | --- |
| Sow_D0 | Chao1 | 1379.86 | 1301.90 | 1475.24 | 1424.00 | 1364.67 | 0.426 |
|  | Ace | 1453.81 | 1388.18 | 1530.27 | 1499.11 | 1412.56 | 0.436 |
|  | Shannon | 5.23 | 5.12 | 5.29 | 5.55 | 4.86 | 0.547 |
|  | Simpson | 0.86 | 0.85 | 0.89 | 0.92 | 0.87 | 0.565 |
| Sow_D21 | Chao1 | 1890.85^a^ | 1456.39^bc^ | 1537.05^b^ | 1486.74^b^ | 1310.89^c^ | 0.000 |
|  | Ace | 2017.90^a^ | 1552.72^bc^ | 1640.46^b^ | 1593.33^bc^ | 1408.34^c^ | 0.000 |
|  | Shannon | 6.82 | 6.61 | 6.88 | 7.01 | 6.95 | 0.252 |
|  | Simpson | 0.97 | 0.96 | 0.97 | 0.98 | 0.97 | 0.155 |
| Piglet_D21 | Chao1 | 1170.00 | 1039.17 | 1156.31 | 1109.57 | 1089.24 | 0.270 |
|  | Ace | 1261.10 | 1119.35 | 1245.66 | 1169.73 | 1160.12 | 0.252 |
|  | Shannon | 6.48^a^ | 5.47^ab^ | 5.80^ab^ | 5.20^b^ | 5.74^ab^ | 0.023 |
|  | Simpson | 0.96^a^ | 0.93^ab^ | 0.93^ab^ | 0.90^b^ | 0.96^a^ | 0.002 |

Control (CON, basal diet), treatment group 1 (TRT1, basal diet + 2 kg/t BZP), treatment group 2 (TRT2, basal diet + 1 kg/t QZGSP), treatment group 3 (TRT3, basal diet + 2 kg/t QZGSP), and treatment group 4 (TRT4, basal diet + 3 kg/t QZGSP).

The same lowercase letters indicate no statistically significant difference (*p* > 0.05), while different lowercase letters indicate a statistically significant difference (*p* < 0.05).
