# Supplementary material for: Effects of supplemental feeding of Chinese herbal mixtures to perinatal sows on antioxidant capacity and gut microbiota of sows and their offspring piglets
Source: Front Microbiol. 2024 Sep 12;15:1459188. doi: 10.3389/fmicb.2024.1459188 (PMC11424466; doi:10.3389/fmicb.2024.1459188)
Supplement: Supplementary file 1 [file Data_Sheet_1.ZIP › Supplementary files/Data sheet 4.zip!/Supplementary files 3 Adonis similarity analysis results/Sow_D0/adonis.TRT1_TRT4.pdf]

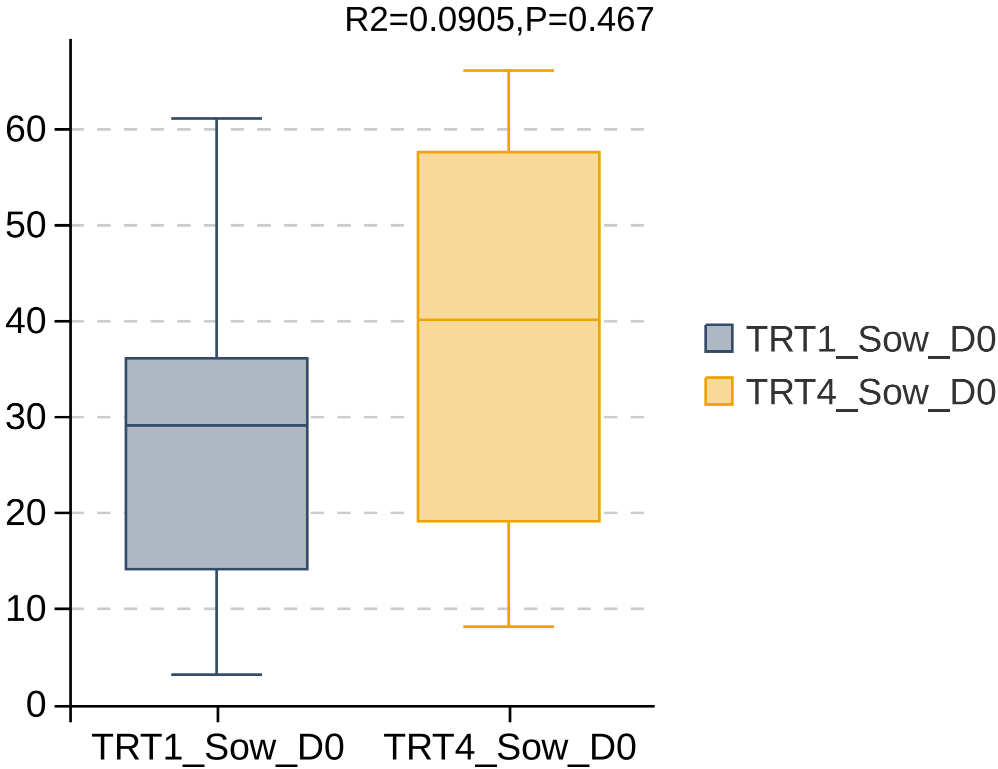

R2=0.0905,P=0.467
60
50
40
30
20
10
0
TRT1_Sow_D0
TRT4_Sow_D0
TRT1_Sow_D0
TRT4_Sow_D0
